# The Role of MicroRNAs in the Regulation of K^+^ Channels in Epithelial Tissue

**DOI:** 10.3389/fphys.2015.00352

**Published:** 2015-12-01

**Authors:** Elliot Pilmore, Kirk L. Hamilton

**Affiliations:** Department of Physiology, Otago School of Medical Sciences, University of OtagoDunedin, New Zealand

**Keywords:** miRNA-7, miRNA-194, miRNA-204, miRNA-205, miRNA-802, Kir1.1, Kir2.1, Kir7.1

## Abstract

Our understanding of the modulation of proteins has shifted in direction with the discovery of microRNAs (miRs) over twenty years ago. MiRs are now in the “limelight” as these non-coding pieces of RNA (generally ~22 nucleotides long) result in altered translation and function of proteins. Indeed, miRs are now reported to be potential biomarkers of disease. Epithelial K^+^ channels play many roles in electrolyte and fluid homeostasis of the human body and have been suggested to be therapeutic targets of disease. Interestingly, the role of miRs in modulating K^+^ channels of epithelial tissues is only emerging now. This minireview focuses on recent novel findings into the role of miRs in the regulation of K^+^ channels of epithelia.

## Introduction

Epithelial K^+^ channels perform numerous physiological roles in K^+^ homeostasis of the body. K^+^ channels participate in maintenance of cellular membrane potential, secretion of ions and fluid, maintenance of blood pressure, cell proliferation, and renal fibrosis (Balut et al., 2012). Indeed, many epithelia K^+^ channels contribute to disease; therefore, it is not surprising that K^+^ channels have been identified as potential therapeutic targets (Wulff et al., 2009; Wulff and Köhler, 2013). MicroRNAs are gaining importance as therapeutic targets for disease (Tutar et al., 2015). Surprisingly, there is a significant gap in our knowledge of the role of miRs in modulating epithelial K^+^ channels. This minireview focuses on recent novel findings into the role of miRs in the regulation of K^+^ channels of epithelia.

## Formation of miRNAs

MiRs are short non-coding pieces of RNA, which are ~22 nucleotides long. Ambros and colleagues (Lee et al., 1993) described the first miR identified in *Caenorhabditis elegans* when they isolated the *lin-4* gene. They reported that *lin*-4 did not code for a protein, instead it produced a short non-coding piece of RNA which contained semi-complimentary sequences to multiple areas in the 3′-untranslated region (UTR) of *lin-14* mRNA (Lee et al., 1993). Indeed, Ambros and co-workers suggested that *lin*-4 regulated the translation of the Lin**-14 protein by an antisense RNA-RNA interaction. As of this writing, the microRNA database lists >28,600 loci of miRNAs (Kozomara and Griffiths-Jones, 2014; http://www.mirbase.org/).

**Figure 1:**
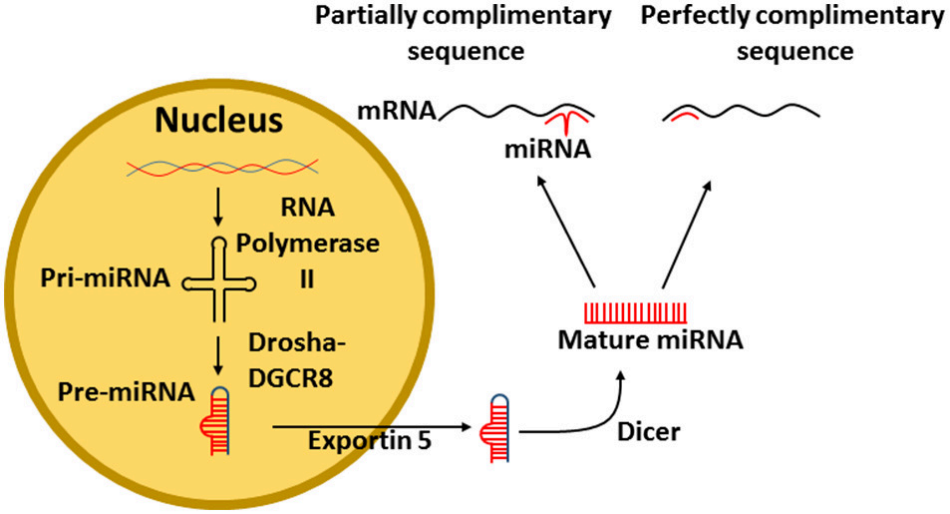
**Transcription of miRNA from DNA**. Pri-miRNA is transcribed from DNA by the enzyme RNA polymerase II. The pri-miRNA is recognized by DGCR8 that associates with Drosha which cleaves the pri-miRNA to form a pre-miRNA, The pre-miRNA exits the nucleus via exportin 5. Finally, the pre-miRNA is cleaved by Dicer into mature miRNA. The mature miRNA can then bind either perfectly or imperfectly to its target mRNA. DGCR8, DiGeorge syndrome critical region 8.

MiRs are transcribed in the nucleus of cells from DNA. The enzyme RNA polymerase II (RNase II) transcribes DNA into a primary RNA (pri-miRNA) within the nucleus. The pri-miRNA is recognized by the nuclear protein DiGeorge syndrome critical region 8 (DGCR8) that associates with Drosha, a RNase III, which cleaves the pri-miRNA generating a precursor miR-RNA (pre-miRNA) (Figure 1). The pre-miRNA then exits the nucleus via a nuclear pore with the assistance of Exportin 5 (Yi et al., 2003). Finally, the pre-miRNA is cleaved by the enzyme Dicer, resulting in the mature miRNA (Figure 1) (Filipowicz et al., 2005).

Mature miRNAs bind to the 3′UTR of mRNA to repress gene expression (Filipowicz et al., 2005), however, modulation of some miRs results in altered protein upregulation (Bhattacharyya et al., 2006). It is apparent, that miRs have differing effects, depending on how they bind to their target mRNA. If the miR sequence is perfectly complimentary (Figure 1), then the miR will lead to the degradation of the mRNA (Jing et al., 2005). However, if the miR sequence is only partially complimentary to its target mRNA, only part of the miR will bind to the mRNA, resulting in blocked protein formation (Lim et al., 2005).

## MiR-802 and miR-194 increase K_IR_1.1 (KCNJ1) abundance in the kidney by indirect pathways

In the cortical collecting duct (CCD), K_IR_1.1 aids in regulating the amount of K^+^ in the body by selectively secreting K^+^ into the urine (Welling and Ho, 2009). Indeed, the modulation and distribution of K_IR_1.1 in the plasma membrane is altered by dietary K^+^ intake (Wang, 2004). Recent studies have established that miRs participate in the regulation of K_IR_1.1, and are also, in part regulated by K^+^ intake (Lin et al., 2011, 2014).

## miR-802 and K_IR_1.1

**Figure 2:**
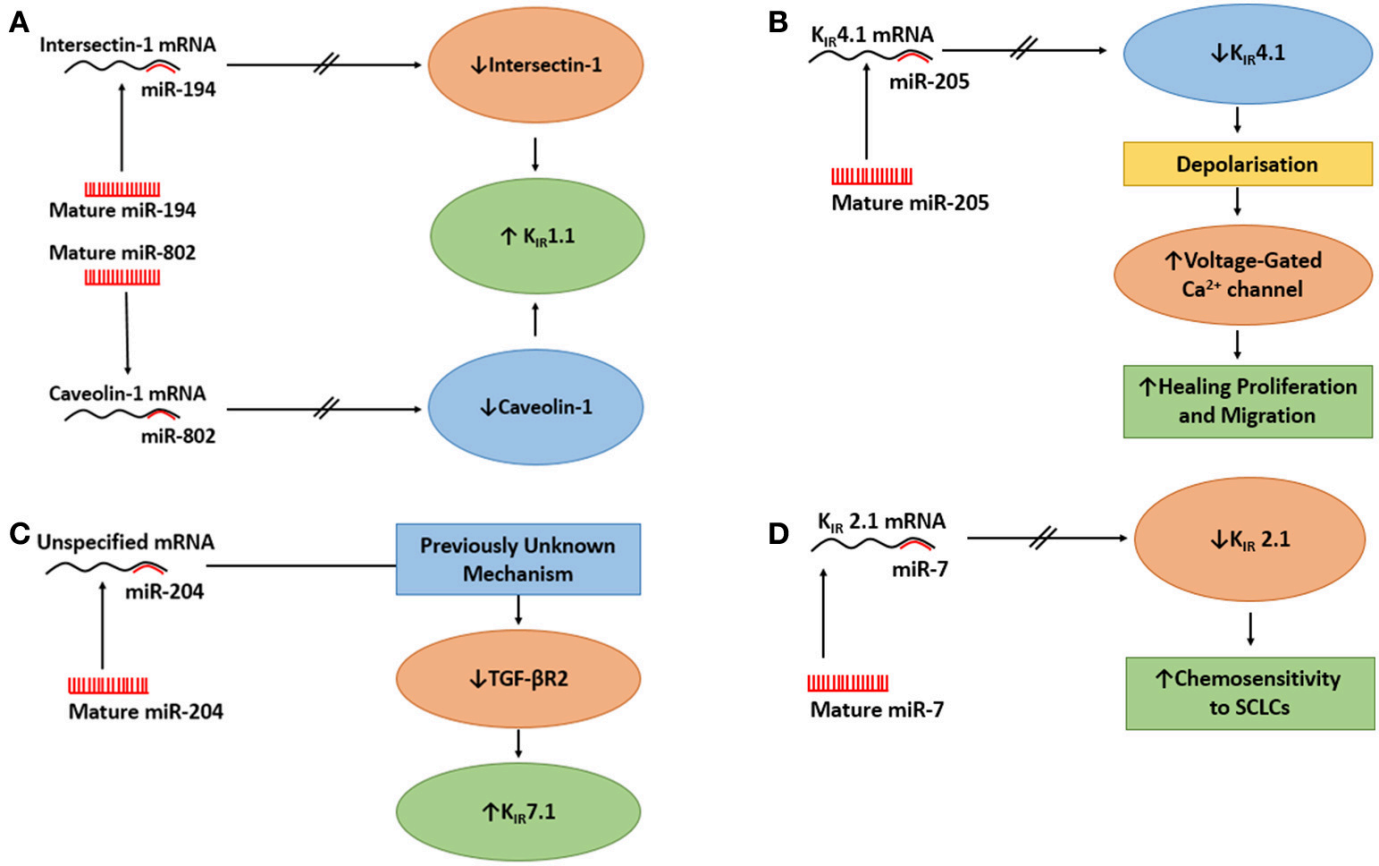
**The actions of miRs on epithelial K^+^ channels**. **(A)** The action of miR-194 and miR-802 on upregulation of K_IR_1.1. Upper Panel: MiR-194 inhibits Intersectin 1, which mitigates the inhibition of K_IR_1.1 by intersectin 1 and increases K_IR_1.1. Lower Panel: miR-802 inhibits caveolin 1 which relieves the inhibition of K_IR_1.1 by caveolin 1 and increases K_IR_1.1. **(B)** The action of miR-205 on K_IR_4.1 in a native human corneal epithelial cells (HCECs) it to suppress K_IR_4.1 causing the cells to depolarize, which activates voltage-gated Ca^2+^ channels in HCECs, thus, increasing the healing process. **(C)** The action of miR-204 on K_IR_7.1 in retinal pigment epithelium. The effect of miR-204 on the increased expression of K_IR_7.1 was caused by miR-204's suppressing action on TGF-βR2, through an unknown mechanism, followed by reduced signaling of protein kinase C which resulted in increased expression of K_IR_7.1. **(D)** MiR-7 regulates the expression of K_IR_2.1 in small-cell lung cancer cells (SCLCs). When miR-7 levels was elevated, the mRNA for K_IR_2.1 was reduced which reduced the expression of K_IR_2.1 at the membrane. There was a inverse correlation between MiR-7 expression levels and the expression of K_IR_2.1 and multi-drug resitance protein 1 which resulted in increased chemosenetivity to SCLCs.

Wang and colleagues (Lin et al., 2011) provided the first evidence that mirR-802 regulated membrane expression and activity of K_IR_1.1 by modulating caveolin-1 (Figure 2A). Initially, the authors performed a miR microarray assay on mouse kidney of animals fed a high K^+^ diet to identify potential miRs that might modulate K_IR_1.1. One miR identified was miR-802. The authors used multiple approaches to determine the role of miR-802 in the regulation of K_IR_1.1. Using Northern blot and PCR experiments, they demonstrated that miR-802 was elevated in the kidney of mice fed a high K^+^ diet. Additionally, using qRT-PCR, they reported increased levels of pre-miR-802 in CCDs isolated from mice fed a high K^+^ diet. Therefore, Lin et al. (2011) established that miR-802 was present in the mouse CCDs and the miR was modulated by high K^+^ diet.

After which, Lin et al. (2011), used databases and identified that the 3′UTR of caveolin-1 contained a recognized binding site for miR-802. Caveolin-1 is a scaffolding protein located in the plasma membrane of most cells (Li et al., 1996; Schubert et al., 2002). The authors used human embryonic kidney (HEK) cell line, a caveolin-1 mutant 3′UTR and a microRNA-sponge (to “absorb” the mature form of miR-802) approach. They demonstrated that miR-802 modulated the 3′UTR of caveolin-1 (luciferase activity) and the miR-802 “sponge” increased the expression of endogenous cavelolin-1 (immunoblot), providing evidence that miR-802 reduced expression of caveolin-1. Since miR-802 regulated the expression of caveolin-1, they hypothesized that a high K^+^ diet would result in reduced caveolin-1 expression. Indeed, they provided conclusive immunoblot evidence that caveolin-1, but not caveolin-2, was reduced in both the kidney of the mice and rats fed a high K^+^ diet.

Thereafter, Lin et al. (2011) turned their efforts to linking miR-802 and caveolin-1 in the regulation of K_IR_1.1. Initially, they asked whether caveolin-1 and K_IR_1.1 were closely associated in a microdomain. They used a detergent-free purification technique to extract caveolin-1 and K_IR_1.1 from the mouse kidney and analyzed extracts in a parallel centrifugation continuous sucrose gradient. They demonstrated that K_IR_1.1 was located in caveolin-1 rich fractions suggesting caveolin-1 and K_IR_1.1 were physically close. Second, they identified three presumed caveolin-1 binding motifs in the N-terminus of K_IR_1.1, after which, they demonstrated, in HEK cells by immunoblot, that transfected K_IR_1.1 was co-immunoprecipitated with endogenous caveolin-1, and that caveolin-1 was co-immunoprecipitated with the K_IR_1.1 N-terminus.

As mentioned above, caveolin-1 is a scaffolding protein that regulates endocytosis and exocytosis of surface proteins (Wyse et al., 2003; González et al., 2007). Lin et al. (2011) asked whether caveolin-1 regulated the surface expression of K_IR_1.1. They used a surface biotin-labeling technique with M-1 cells (CCD cell line, Stoos et al., 1991) that were transfected with GFP- K_IR_1.1 to examine the effect of caveolin-1 on K_IR_1.1 expression. After 48 h, they demonstrated caveolin-1 reduced the surface expression of K_IR_1.1 as measured by immunoblot. Subsequently, reducing endogenous caveolin-1 with siRNA resulted in increased expression of K_IR_1.1 at the plasma membrane. These data clearly demonstrated that caveolin-1 regulated the expression of K_IR_1.1 in M-1 cells. Further, to determine if the expression of caveolin-1 reduced the amount of K_IR_1.1 at the membrane, the authors provided functional data, with patch-clamp experiments, that co-expression of caveolin-1 and K_IR_1.1 caused a large decrease in K^+^ current when compared to K^+^ current of cells not transfected with caveolin-1. Finally, with a combination of perforated whole cell experiments with HEK cells, Lin et al. (2011) reported that co-transfection of K_IR_1.1 and pre-miR-802 or K_IR_1.1 + pre-miR-802 + caveolin-1 for 24 h resulted in that (i) pre-miR-802 increased K^+^ currents of K_IR_1.1, (ii) the effect of miR-802 on K^+^ currents was due to decreased expression of caveolin-1, since expression of mutant caveolin-1 (missing 3′UTR) reduced the effect of pre-miR-802 and decreased the K^+^ currents, and (iii) in M1-cells, miR-802 stimulated the surface expression of K_IR_1.1. Therefore, miR-802 increased the surface expression of K_IR_1.1, by reducing caveolin-1 that increased the activity of K_IR_1.1 (Figure 2A).

## miR-194 and K_IR_1.1

MiR-194 is present in kidney (Tian et al., 2008). As with miR-802, Wang and coworkers (Lin et al., 2014) used a similar high K^+^ diet experimental approach to investigate the role of miR-194 in the regulation of K_IR_1.1 by targeting intersectin 1 (ITSN1). ITSN1 is a cytoplasmic membrane-associated protein that aids in trafficking of endosomes (Yamabhai et al., 1998; Okamoto et al., 1999).

Wang and colleagues (Lin et al., 2014) demonstrated the up regulation of miR-194 in the mouse kidney of animals fed a high K^+^ diet as determined by Northern blot. Next, they demonstrated, by qRT-PCR, that miR-194 was increased in the CCDs of mice that were fed a high K^+^ diet. The authors then, identified, through database analysis, that the 3′UTR of ITSN1 contained a putative binding site for miR-194. Therefore, they hypothesized if K^+^ diet altered expression of ITSN1 through miR-194, then, a high K^+^ diet should reduce expression of ITSN1. Indeed, high dietary K^+^ reduced the expression of ITSN1 in the mouse kidney. Based on those results, they examined whether miR-194 regulated the expression of ITSN1 by using a wild-type ITSN1-3′UTR, mutant ITSN1-3′UTR, and a luciferase assay approach. Co-expression (into HEK293T cells) of miR-194 and ITSN1-3′UTR, but not mutant ITSN1-3′UTR resulted in altered luciferase activity providing evidence that miR-194 regulated ITSN1. To verify that the effect of miR-194 on ITSN1 expression was due to ITSN1-3′UTR, the authors used a flag-tagged ITSN1-3′UTR and 3′UTR-free ITSN1 immunoblot approach with HEK293T cells. MiR-194 reduced expression of ITSN1-3′UTR but had no effect on the expression of 3′UTR-free ITSN1. These data demonstrated that miR194 modulated ITSN1 via the 3′UTR.

He et al. (2007) had previously shown that ITSN1 regulated the activity of K_IR_1.1 by up-regulating With-No-Lysine (WNK)–induced endocytosis of K_IR_1.1. Therefore, they predicted that miR-194 should alter K_IR_1.1 channel activity by regulating ITSN1. By using perforated whole cell patch experiments of HEK293T cells, the authors reported that co-transfection of K_IR_1.1 and pre-miR-194 increased the K^+^ current compared with control cells only expressing K_IR_1.1. In order to determine if miR-194 increased expression of K_IR_1.1 at the membrane by modulation of ITSN1, they used biotin-labeling to determine the surface expression of K_IR_1.1. Therefore, K_IR_1.1 expressing HEK293T cells were transfected with pre-miR-194, pre-miR-194 + ITSN1 or a control oligonucleotide. Immunoblot results demonstrated that pre-miR-194 enhanced K_IR_1.1 surface expression compared to the control nucleotide and that ITSN1 prevented any increase in K_IR_1.1 surface expression. The authors concluded that miR-194 increased K_IR_1.1 channel activity by enhancing the surface expression of the channel as a result of miR-194 decreasing ITSN1-WNK-induced endocytosis of K_IR_1.1, as demonstrated by co-expression of ITSN1 and miR-194; which reversed the effect of miR-194 on K_IR_1.1 surface expression (Figure 2A).

## miR-205 suppresses K_IR_4.1 (KCNJ10) in corneal epithelial cells

K_IR_4.1 is an inwardly rectifying K^+^ channel cloned from heart, brain, and skeletal muscle (Bond et al., 1994). K_IR_4.1 has been demonstrated in many epithelial tissues including the cornea (Kofuji et al., 2000; Hamilton and Devor, 2012). K_IR_4.1 plays roles in cell adhesion-migration, cell proliferation, and apoptosis by modulating membrane potential (Chen and Zhao, 2014; Wang et al., 2014). There is a dearth of information about the direct evaluation of K^+^ channels in the healing of differentiated epithelial cells (Girault and Brochiero, 2014) or the effect of miRs on the action of K_IR_4.1.

Lin et al. (2013) provided the first evidence of a miR that modulated K_IR_4.1 in the healing process after injury in human corneal epithelial cells (HCECs). They observed that when a scratch injury was applied to HCECs, miR-205 expression was elevated, but miR-16, another expressed miR in HCECs, was not altered as determined by qRT-PCR. Indeed, they demonstrated that miR-205 agomir stimulated cell migration in wound closure of HCECs, while miR-205 antagomir did not. The authors tested their hypothesis that miR-205 stimulated wound healing by reducing K_IR_4.1 by examining the effect of barium, a K^+^ channel blocker, on HCECs transfected with miR-205 antagomir in the absence and presence of barium. Cells treated with barium increased wound recovery compared with cells transfected with miR-205 antagomir, alone, which had a slower recovery. It should be noted that barium is a generic K^+^ channel inhibitor; nortriptyline has been used to inhibit K_IR_4.1 in astrocyte cells (Su et al., 2007). These data suggested that miR-205 altered K_IR_4.1 expression. Knockdown of K_IR_4.1, by siRNA, increased the growth rate of wound injury suggesting reduced K_IR_4.1 activity increased cell regrowth.

Since, down regulation of K_IR_4.1 enhanced cell regrowth, Lin and coworkers hypothesized that miR-205 may modulate K_IR_4.1 in HCECs. In order to test this, they identified a potential binding region of miR-205 in the 3′UTR of K_IR_4.1. Therefore, using their dual luciferase reporter assay (Lin et al., 2011), they demonstrated that miR-205 decreased K_IR_4.1 in wild type K_IR_4.1-3′UTR in HCECs compared to cells with K_IR_4.1 and mutant K_IR_4.1-3′UTR. Then, the authors reported, with scratch wound experiments, that the K_IR_4.1 expression was reduced after 24 h, while cells transfected with miR-205 antagomir increased the expression of K_IR_4.1. This further verified that miR-205 modified the expression of K_IR_4.1. Lastly, the authors used the patch-clamp technique and determined that miR-205-antagomir increased K^+^ currents while miR-205 agomir reduced K^+^ currents of HCECs and that these currents were characteristic of K_IR_4.1 (Takumi et al., 1995).

Thus, Lin et al. (2013) suggested that following scratch injury of HCECs, there was down regulation of K_IR_4.1 by miR-205 that caused the cells to depolarize more rapidly, which lead to increased activation of voltage-gated Ca^2+^ channels (Lin et al., 2013) increasing the healing process (Figure 2B). However, it would have been prudent if the authors had conducted experiments testing the effects of altering the function of voltage-gated Ca^2+^ channels while examining the expression of K_IR_4.1 and miR-205 levels. However, increased intracellular Ca^2+^ in HCECs has been suggested to be essential for the release of growth factors or cytokines to initiate cell proliferation in the cornea (Du et al., 2006).

## miR-204 indirectly suppresses K_IR_7.1 (KCNJ13) in retinal pigment epithelium

K_IR_7.1 is expressed in retinal pigment epithelium (RPE) and facilitates interactions between the RPE and photoreceptors during transitions between light and dark (Wang et al., 2010). MiR-204 was reported in high amounts in RPE of mice (Bak et al., 2008). Wang et al. (2010) have provided the first report of miR-204 in the modulation of K_IR_7.1 in the RPE.

Initially, Wang et al. (2010) conducted a miRNA expression profile in native human fetal RPE (hfRPE) by qRT-PCR and identified that miR-204 was a highly enriched miR. They further identified miR-204 in fetal RPE culture and native fetal retina and RPE by Northern blot. Little is known about the physiological role of miR-204 and coupled with a high expression of K_IR_7.1 in the RPE (Yang et al., 2008), Wang et al. (2010) examined if miR-204 regulated K_IR_7.1. Therefore, they conducted semi-quantitative immunoblot experiments in which they transfected hfRPE with anti-miR-204 or anti-miR-negative control oligonucleotide and probed for K_IR_7.1. They demonstrated that anti-miR-204 reduced the expression of K_IR_7.1 compared with control cells, thus suggesting that K_IR_7.1 is regulated by miR-204. Wang et al. (2010) identified that the 3′UTR of transforming growth factor - beta receptor 2 (TGF-βR2) was a potential target of miR-204. Using a luciferase approach, the authors transfected HEK cells with miR-204 mimic and either wt-TGF-βR2-3′-UTR or mutant-TGF-βR2-3′-UTR. miR-204 mimic reduced the luciferase activity for only wt-TGF-βR2-3′-UTR. Further, they confirmed, with an anti-miR-204 approach as described above, that anti-miR-204 increased the expression of TGF-βR2 of the hfRPE. From their data, Wang et al. (2010) proposed that the effect of miR-204 on the increased expression of K_IR_7.1 was caused by miR-204's suppressing action on TGF-βR2 followed by reduced signaling of protein kinase C which resulted in increased expression of K_IR_7.1 as noted by others (Zhang et al., 2008; Figure 2C).

## Role of miR-7 in regulation of K_IR_2.1 (KCNJ2) in small-cell lung cancer cells (SCLCs)

K_IR_2.1 is an inward rectifying K^+^ channel that was described by Jan and colleagues (Kubo et al., 1993). K_IR_2.1 maintains the resting membrane potential in numerous cell types including SCLCs (Sakai et al., 2002; Hibino et al., 2010). Jirsch et al. (1993) demonstrated that expression of inwardly rectified K^+^ channels was enhanced in the presence of multidrug resistance-associated protein.

Recently, Liu et al. (2015) provided a link between miR-7 and the upregulation of K_IR_2.1 in the modulation of multidrug resistance of SCLCs. They demonstrated that expression of K_IR_2.1 was significantly associated with clinical stage and chemotherapy response in patients with SCLC. Further, they reported that K_IR_2.1 expression was more common at serious disease stage and in drug-resistant patients than in limited disease stage patients or in drug-sensitive patients. Having demonstrated a link between K_IR_2.1 and multidrug resistance, the authors focused their effort, using immunoblot and Co-IP experiments, and established that K_IR_2.1 increased the expression of multi-drug resistance protein 1 (MRP1) and that these proteins interacted.

MiR-7 plays an integral part in initiation, proliferation, invasion, survival, and death by targeting oncogenic signaling pathways (Gu et al., 2015). Next, Lui and colleagues hypothesized that the high expression of K_IR_2.1 might be regulated by endogenous miR-7. Indeed, they identified, that miR-7 had a potential interaction site in the 3′UTR of K_IR_2.1. Using a luciferase reporter approach, they transfected H69 cells (human SCLC cell line) with either K_IR_2.1-3′UTR-wt, K_IR_2.1-3′UTR-mutant, or control vector with miR-7 agomir or antagomir or negative control vector. There was suppressed luciferase activity when the miR-7 agomir was cotransfected with K_IR_2.1-3′UTR-wt, but not when K_IR_2.1-3′UTR-mutant was cotransfected with either MiR-7 agomir or antagomir, suggesting that Kir2.1 is a direct target of miR-7 in SCLCs. The authors examined the effect of miR-7 on chemoresistance of SCLCs by analyzing the sensitivity of SCLCs to chemotherapeutic drugs (adriamycin, cisplatin, and eroposide) after the transfection of miR-7 agomir, antagomir, or negative control vector. Their results indicated that upregulation of miR-7 sensitized SCLCs to all drugs, while downregulation of miR-7 desensitized SCLCs. These data suggested that miR-7 downregulation may explain the effects of K_IR_2.1 on the chemoresistance of SCLCs. Lastly, the authors confirmed the association between the expression of K_IR_2.1 and miR-7 by analyzing the miR-7 expression, by qRT-PCR, in 52 human SCLC tissue specimens. Correlation data demonstrated that miR-7 expression was inversely correlated to K_IR_2.1 and MRP1 expression. Additionally, low-level expression of miR-7 was significantly seen with a more aggressive clinical stage of SCLC. Indeed, SCLC patients with low levels of miR-7 expression exhibited shorter survival times than patients with high miR-7 expression. In summary, Liu et al. (2015) provided a novel method in which K_IR_2.1 and miR-7 regulate the sensitivity of SCLC to chemotherapeutic drugs possibly through the regulation of MRP1 (Figure 2D).

## Conclusions

In this review, we examined the role of miRs in regulating epithelial K^+^ channels. While there is little information available, so far, this is an emerging field of research. The information gained is important, as epithelial K^+^ channels play vital roles in survival and homeostasis.

## Author contributions

KH and EP developed the concept for this mini review together. EP researched the literature for the key papers used in this mini review. KH took a early draft prepared by EP and increased the size of the manuscript considerably. EP drew all of the figures.

### Conflict of interest statement

The authors declare that the research was conducted in the absence of any commercial or financial relationships that could be construed as a potential conflict of interest.
